# Concurrent Zoster Sine Herpete and Hepatosplenic Fungal Infection in a Cancer Patient: A Case Report

**DOI:** 10.7759/cureus.72117

**Published:** 2024-10-22

**Authors:** Emily M Pearson, Guy Handley, John Greene

**Affiliations:** 1 Morsani College of Medicine, University of South Florida, Tampa, USA; 2 Infectious Disease and Internal Medicine, University of South Florida, Tampa, USA; 3 Infectious Diseases and Tropical Medicine, Moffitt Cancer Center, Tampa, USA

**Keywords:** cancer, candida albicans, cystobasidium, hairy cell leukemia, hepatosplenic mycosis, immunocompromised, varicella-zoster virus, zoster sine herpete

## Abstract

In patients undergoing treatment for hematological cancers, like hairy cell leukemia (HCL), appropriate management of infections is of utmost importance. This paper describes the successful treatment of an HCL patient who had both visceral zoster sine herpete (ZSH) and hepatosplenic fungal infection, possibly caused by *Cystobasidium*. To our knowledge, this is the first such case to appear in the literature, as these are rare conditions associated with states of immunosuppression, similar to those observed in hematological malignancy. Nonspecific presenting symptoms and the ineffectiveness of standard blood cultures make diagnosing these infections challenging. Newer tools, such as next-generation sequencing, may help clinicians quickly identify uncommon infections in this high-risk population of immunocompromised individuals. In addition to rare pathogens, healthcare workers must be mindful of infections that may not show typical symptoms, such as the absence of a dermatomal rash in cases of ZSH.

## Introduction

The incidence of infection is known to be high among patients with hairy cell leukemia (HCL) [1]. In a long-term study of HCL patients, 37% developed at least one infection [1]. Standard first-line treatment of HCL involves purine analogs, such as cladribine or pentostatin [2]. Cladribine is structured similarly to purines and is incorporated into the DNA of proliferating cells, triggering cell death [3]. This effectively reduces the number of malignant lymphoid cells and is therefore used to treat hematological cancers like HCL. However, these drugs also decrease the number of nonmalignant immune cells, including lymphocytes [3] and neutrophils [4]. Rituximab, an anti-CD20 monoclonal antibody, may also be added to the treatment regimen, particularly in the case of relapsing disease [2,4].

Successful treatment does not cure the condition but instead decreases the presence of HCL and enables the blood count levels to return to normal [4]. Though long-term survival rates for HCL are generally high [1,5], infections present the greatest cause of mortality among HCL patients [4]. When working with these patients, monitoring for signs of infection and prompt initiation of appropriate treatment are paramount in improving long-term outcomes. Here, we present a case of a patient undergoing treatment for relapsed HCL who was diagnosed with hepatosplenic mycosis, or fungal infection of the liver and spleen, and visceral zoster sine herpete (ZSH). The latter describes viral zoster reactivation in the visceral nervous system without a rash.

## Case presentation

A 66-year-old man with relapsed HCL presented to our hospital with a two-week history of worsening abdominal pain radiating to the right flank. The patient had completed a course of cladribine three months prior as well as three weeks of rituximab therapy at the time of presentation. He had also recently undergone a month-long hospitalization for an acute kidney injury and septic bacteremia due to *Mycobacterium abscessus*. The *Mycobacterium* infection required an extensive antibiotic regimen that he completed three months prior to presentation. Other past medical history included hypertension, chronic mild memory impairment, gait instability, and chronic neck pain managed with opioids.

The patient described severe, stabbing pain exacerbated by movement. He reported sweating, chills, and nausea with an episode of dry heaving. He denied fever, dyspnea, precordial chest pain, headache, numbness, tingling, or rash. He reported use of marijuana and tobacco products. The patient described a history of heavy alcohol use but no alcohol use for the past year. He reported no known allergies.

The vital signs showed a heart rate of 84 beats per minute, a respiratory rate of 16 breaths per minute, blood pressure of 156/95 mmHg, a temperature of 36.4°C, and an oxygen saturation of 96% on room air. On physical exam, palpation of the abdomen was unremarkable. Right costovertebral and right lower paraspinal tenderness were noted, raising suspicion for aortic dissection as the cause of radiating flank pain. 

**Figure 1:**
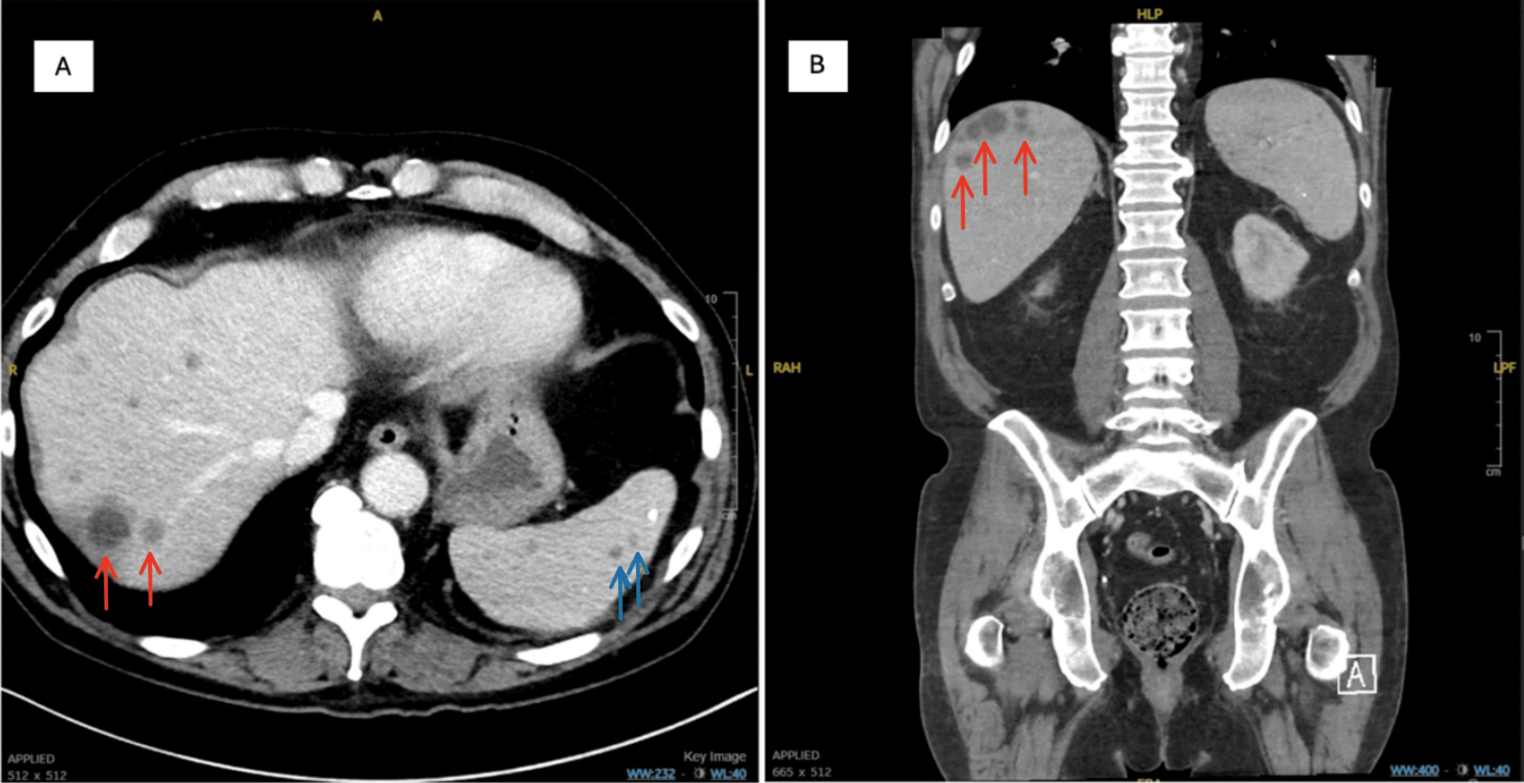
Imaging performed upon patient's initial presentation showing multiple hypoenhancing lesions of the liver (red arrows) and the spleen (blue arrows). CT of the abdomen and pelvis with contrast in axial view (A) and coronal view (B).

Laboratory findings on admission included a white blood cell count of 11.52 K/μL (reference range, 4.0-11.0 K/μL), with an absolute neutrophil count >1.5 K/μL (2.5-7.5 K/μL) and an automated lymphocyte count of 2.01 K/μL (1.0-4.8 K/μL). Laboratory findings also showed alkaline phosphatase (ALP) of 186-193 U/L (44-147 U/L), lactic acid dehydrogenase of 185 U/L (140-280 U/L), aspartate aminotransferase of 16-20 U/L (8-33 U/L), alanine aminotransferase of 15 U/L (4-36 U/L), albumin of 4.0-4.1 g/dL (3.5-5.5 g/dL), and total bilirubin of 0.4-0.5 mg/dL (0.1-1.2 mg/dL). CT of the chest and abdomen showed atherosclerosis of the aorta but no dissection or stenosis. Imaging also showed multiple hypoenhancing cystic lesions on the liver, all less than 3 cm in size, as well as mild splenomegaly with multiple subcentimeter splenic lesions. Enlargement of mediastinal, periportal, and retroperitoneal lymph nodes was noted (Figure 1).

A liver biopsy was performed to determine whether the lesions seen on imaging were leukemic metastases or infectious abscesses. Fungal staining of liver biopsy samples showed the presence of hyphal elements at a moderate level, while gram staining was unremarkable. Additionally, 1,3-β-D-glucan assay of a blood sample was positive at 236 pg/mL (reference range, <80 pg/mL). Though the patient was initially prescribed piperacillin/tazobactam, low suspicion for bacterial infection prompted the discontinuation of this antibiotic. Instead, the patient was switched to voriconazole (300 mg, twice daily) and prophylactic acyclovir.

On the sixth day of admission, plasma samples sent for testing confirmed the presence of both *Candida albicans* (492 molecules per microliter; reference range, <10 molecules/μL) and varicella-zoster virus (VZV) (255,058 molecules per microliter; <10 molecules/μL) through microbial cell-free DNA (mcfDNA) analysis. The dosage of acyclovir was increased from prophylactic levels to high dosages (IV, 10 mg/kg q8h) for two days while inpatient. After an eight-day admission, the patient was discharged home. The day after discharge, results were returned from the polymerase chain reaction (PCR) testing of the blood samples, confirming the presence of VZV viremia. At home, the patient completed a regimen of valacyclovir (PO, 1 g thrice daily) for two weeks, after which he resumed prophylactic acyclovir (PO, 800 mg twice daily) in preparation for continued HCL treatment. Additionally, PCR testing of liver biopsy samples sent to the University of Washington after discharge detected the presence of *Cystobasidium halotolerans* or *Cystobasidium terricola*. Based on these findings, we strongly suspected a *Cystobasidium* pathogen over *C. albicans*, which was suggested only by blood samples and not liver tissue samples. With these results, the prescribed voriconazole treatment was continued for one year after discharge.

**Figure 2:**
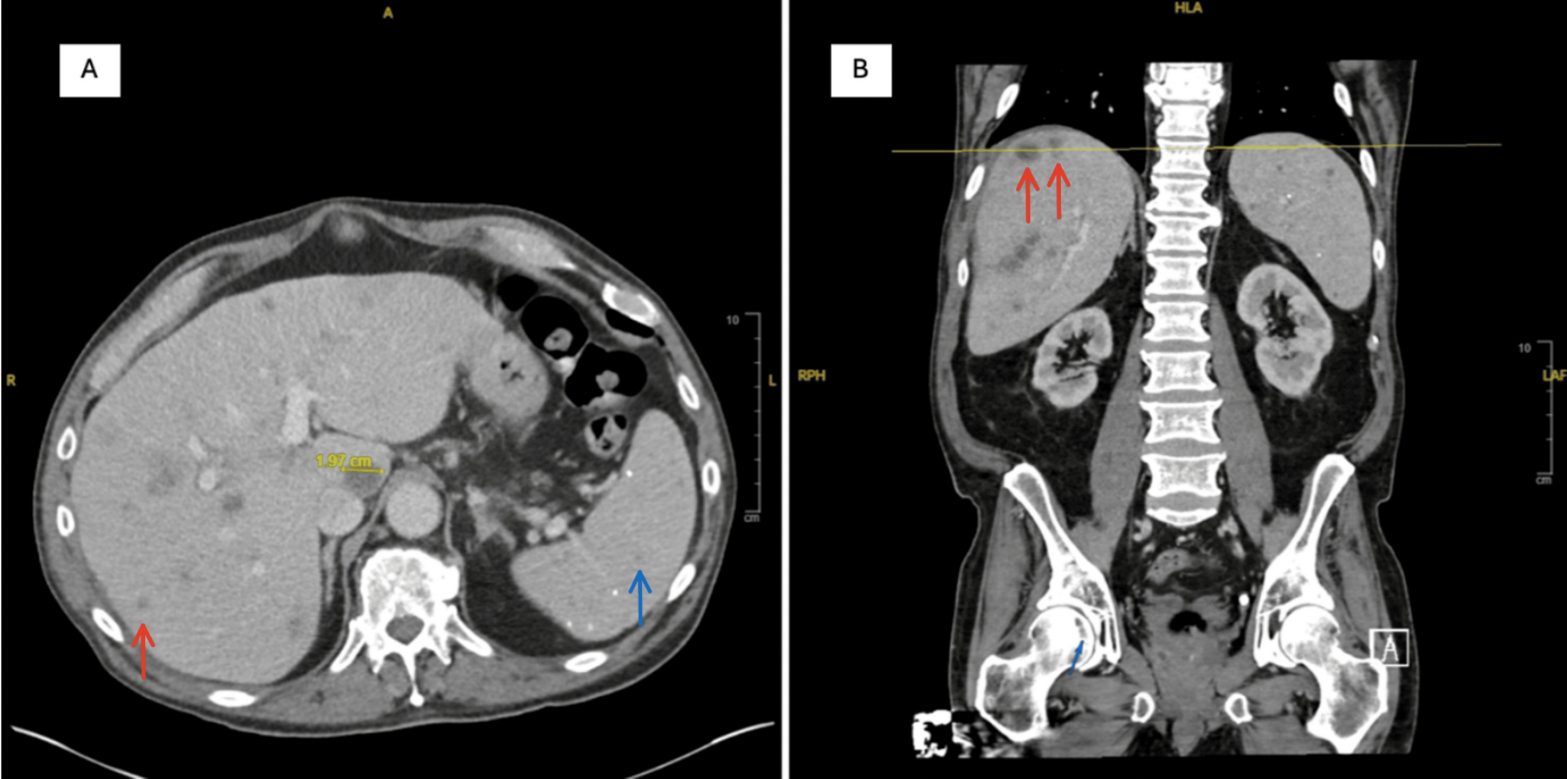
Imaging performed at eight-week follow-up showing improvement of lesions of the liver (red arrows) and the spleen (blue arrow). CT of the abdomen and pelvis with contrast in axial view (A) and coronal view (B).

Continued monitoring of his liver function over the following three months showed rising ALP levels that peaked at 650 U/L four weeks after presentation. However, ALP then decreased to 300 U/L at a two-month follow-up, likely reflecting progress in the treatment of his hepatic fungal infection. Additionally, PCR testing of the blood samples at a one-month follow-up was negative for VZV. At this time, the patient reported persistent but controlled flank pain and had resumed full HCL treatment. An abdominal CT performed eight weeks after presentation showed modest improvement of the liver and spleen lesions (Figure 2).

## Discussion

HCL is a rare kind of mature B-cell lymphoma, accounting for about 2% of non-Hodgkin’s lymphoid neoplasms in the United States [5]. Normally, infections are a presenting symptom that leads to the initial diagnosis of HCL [4]. In other cases, HCL diagnosis occurs after patients present with pancytopenia, as the aberrant proliferation of B cells causes the bone marrow to produce fewer other cell types [4]. Patients lacking functional immune cells have a decreased capacity to defend themselves against pathogens. Subsequent treatment with cladribine or pentostatin can further contribute to the state of immunosuppression [4]. These individuals are at a high risk for infection, which can significantly affect their survival [6], hospitalization patterns, and medical costs [2]. Frequently encountered etiological agents include bacterial pathogens common in all neutropenic patients (e.g., *Staphylococcus aureus*, *Escherichia coli*, and *Pseudomonas aeruginosa*), but less common bacteria, such as atypical mycobacterial species, have been reported as well. In addition, HCL patients are at risk of being infected by viral (e.g., VZV, herpes simplex virus, and *Cytomegalovirus*) and fungal (e.g., *Candida* species, *Aspergillus* species, *Cryptococcus neoformans*, and *Pneumocystis jirovecii*) pathogens [7].

Our patient was initially suspected to have hepatosplenic candidiasis (HSC), or infection of the liver and spleen caused by a *Candida species*. Most cases of HSC occur in the immunosuppressed and are caused by *C. albicans* [8]. Because standard blood cultures are not reliable for detecting deep-seated *Candida* infections, diagnosis often involves a liver biopsy, imaging, and blood analysis for biomarkers such as 1,3-β-D-glucan [8]. Cancer patients undergoing chemotherapy are at particular risk, as cytotoxic therapy can damage the gut epithelium and provide commensal *Candida* species access to the bloodstream [9].

However, results of the PCR analysis of liver samples returned after discharge indicated *Cystobasidium* as the main causative fungal pathogen. Many of these yeast species were previously classified under *Rhodotorula* [10] and produce 1,3-β-D-glucan, though to a lesser extent than *C. albicans* [11]. This is significant as our patient had a high β-glucan level that was initially thought to correlate with invasive candidiasis. In a systematic review of reported infections by the closely related *Rhodotorula* species, many cases occurred in patients with underlying malignancy or other immunocompromised states. Among the 248 cases accounted for in the review, only two featured liver infections caused by *Rhodotorula* pathogens [12]. Significantly, fluconazole is known to be ineffective against *Rhodotorula* pathogens [12]. In our case, progress toward resolution of the lesions as tracked by CT imaging suggests that voriconazole was an appropriate treatment for this patient’s invasive fungal disease.

The VZV is a human neurotropic virus known to primarily cause varicella, or “chickenpox,” in children and then establish long-term latency in the ganglia along the neuroaxis [13]. Reactivation of the virus can occur as age increases and/or immune function decreases [14], most commonly resulting in the classic zoster rash. ZSH is a rare manifestation of VSV reactivation, wherein the patient experiences a neurological disease without the telltale rash, making the diagnosis challenging [13]. In our case, the patient was suspected to have visceral zoster, in which the virus reactivates in the enteric nervous system and may not cause classic dermatological changes [15,16]. Patients with visceral zoster are often immunocompromised [16] and present with epigastric pain radiating to the back [15], as seen in our case.

Though our patient’s epigastric and flank pain could have been attributed solely to hepatic mycosis, further workup with mcfDNA testing of plasma samples showed a relatively high concentration of VZV, followed by a confirmation of viremia via PCR testing the next day. Based on existing research with PCR-based methods, VZV DNA is only detected infrequently and at low viral loads in blood samples from asymptomatic individuals [17], supporting the idea that our patient had an active, symptomatic VZV reactivation. He was diagnosed with visceral ZSH and never developed any cutaneous manifestations of VZV reactivation throughout the course of his disease. While the incidence of this atypical VZV manifestation is unknown, ZSH is likely underdiagnosed [16] and should be considered by clinicians when working with the immunosuppressed.

In our case, mcfDNA testing from Karius® identified the need for antiviral treatment before traditional PCR-based analysis of blood samples. Available since 2017 [18], the Karius assay provides a noninvasive means of quickly identifying a wide variety of pathogens, including species that are rare or difficult to otherwise detect [19]. Additionally, Karius testing provides fast results [18], and in our case, it prompted escalation of acyclovir therapy sooner than PCR analysis. Given the need to monitor for a variety of possible infections among HCL patients, the Karius assay and other forms of next-generation sequencing may provide an important tool for prompt identification of pathogens that are rare or manifesting atypically among these individuals. However, such assays are also likely to detect commensal microbes in addition to new pathogens. In our case, the assay identified *C. albicans*, but subsequent PCR analysis of the infected tissue revealed *Cystobasidium *as the more likely fungal pathogen. Of note, the Karius Pathogen List does not include *Cystobasidium *or *Rhodotorula* species other than *Rhodotorula mucilaginosa* as of September 2024 [20]. Therefore, the current diagnostic capacity of this service is extensive but not exhaustive, and in our case, multiple techniques may be needed to augment detection capability and reach a final diagnosis.

## Conclusions

Given the significant impact that infections can have on the outcomes for HCL patients, healthcare providers must promptly identify and treat infections in these individuals. Because HCL patients often present in an immunosuppressed state, they are at high risk for a variety of infections that can critically alter the course of their cancer treatment. Our case illustrates how immunosuppressed patients may present with multiple uncommon infections that require vigilant attention. With the mcfDNA analysis through Karius and PCR analysis through the University of Washington, we identified two pathogens that matched the histopathology of patient samples, which could not be identified with traditional culture techniques. Particularly, in cases of intracellular pathogens like VZV or deep-seated fungal infections like hepatosplenic mycosis, molecular techniques may provide an advantage over traditional ones, in terms of sensitivity and speed. Further development and availability of next-generation sequencing tools are needed for the quick detection of rare and covert pathogens, as well as concurrent infections. These diagnostic improvements will allow for earlier initiation of appropriate antimicrobial therapy and enhance outcomes among HCL patients and others susceptible to infection.
